# Therapeutic effects of curcumin supplementation on liver enzymes of nonalcoholic fatty liver disease patients: A systematic review and meta‐analysis of randomized clinical trials

**DOI:** 10.1002/fsn3.4144

**Published:** 2024-12-01

**Authors:** Armin Ebrahimzadeh, Anahita Ebrahimzadeh, Sara Fooladshekan, Shokouh Mohseni, Abbas Mohtashamian, Siavash Babajafari, Zahra Sohrabi

**Affiliations:** ^1^ Nutrition Research Center, School of Nutrition and Food Sciences Shiraz University of Medical Sciences Shiraz Iran; ^2^ Dental Research Center Golestan University of Medical Sciences Gorgan Iran; ^3^ Student Research Committee, Department of Nutrition, Faculty of Medicine Kashan University of Medical Sciences Kashan Iran

**Keywords:** alanine aminotransferase, alkaline phosphatase, aspartate aminotransferase, curcumin

## Abstract

Curcumin, as an antioxidant agent, has been proposed as a potential treatment for nonalcoholic fatty liver disease (NAFLD). The aim of the current systematic review and meta‐analysis was to summarize earlier findings regarding the effect of curcumin supplementation on liver enzymes and ALP in NAFLD patients. All studies published up to November 18, 2022, were searched through the PubMed, SCOPUS, and Web of Science databases to collect all randomized clinical trials (RCTs) on NAFLD patients in which curcumin was used as a treatment. A random‐effects model was used to measure pooled effect sizes. Weighted mean differences (WMDs) and 95% confidence intervals (CIs) were used to report pooled effect sizes. Subgroup analysis was utilized to investigate heterogeneity. A total of 14 studies were included in this systematic review and meta‐analysis. Our pooled meta‐analysis indicated a significant decrease in alanine aminotransferase (ALT) following curcumin therapy by pooling 12 effect sizes (WMD: –8.72; 95% CI: –15.16, –2.27, *I* ^2^ = 94.1%) and in aspartate aminotransferase (AST) based on 13 effect sizes (WMD: –6.35; 95% CI: –9.81, –2.88, *I* ^2^ = 94.4%). However, the pooled analysis of five trials indicated that there was no significant association between curcumin therapy and alkaline phosphatase (ALP) in NAFLD patients (WMD: −4.71; 95% CI: −13.01, 3.58, *I* ^2^ = 64.2%). Nevertheless, subgroup analyses showed significant effects of curcumin on ALP with a longer duration of supplementation. The findings of this systematic review and meta‐analysis support the potential effect of curcumin on the management of NAFLD. Further randomized controlled trials should be conducted in light of our findings.

## Graphical abstract

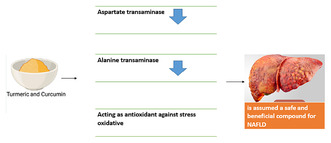

## 1 INTRODUCTION

Nonalcoholic fatty liver disease (NAFLD) has become an increasingly significant public health problem, with a prevalence of approximately 25% of the global population. The highest prevalence has been recorded in the Middle East and South America (Younossi et al., 2016). Despite being common chronic diseases, such as hypertension (30.0–32.2%) and diabetes (22.9%–37.5%), the epidemic scale of NAFLD continues to increase (Estes et al., 2018; Guariguata et al., 2014; Mills et al., 2016). The consequences of NAFLD are not only associated with worsening liver problems but also with metabolic disease, cardiovascular disease, certain tumors, and other illnesses (Adams et al., 2017; Byrne & Targher, 2015; Cai et al., 2019; Rinella, 2015; Sporea et al., 2018). The complications of NAFLD have become a significant burden on the healthcare system (Cai et al., 2019; Chedid, 2017; Cholankeril et al., 2017; Estes et al., 2018; Perumpail et al., 2017).

Some liver enzymes, including AST and ALT, are used to assess liver function (Zou et al., 2020). In some studies, an association between ALT and NAFLD has been observed. Additionally, ALT levels within the normal reference range have sometimes been associated with the risk of NAFLD (Oh et al., 2011; Younossi et al., 2016). The ratio of AST/ALT is used to assess the degree of hepatic steatosis and hepatic fat accumulation (Long et al., 2016; Nanji et al., 1986). However, few studies have evaluated the relationship between the ALT/AST ratio and NAFLD (Zou et al., 2020). Although ALP levels are sometimes slightly increased, it is rarely the only liver function test abnormality (Pantsari & Harrison, 2006).

Various therapeutic practices have been used for patients with NAFLD (Arab et al., 2014; Farhangi et al., 2022). Some herbs and medicinal plants are commonly used for preventing or treating diseases (Mohammadi‐Sartang et al., 2018). However, the long‐term safety and efficacy of these therapies have not yet been fully established (Arab et al., 2014). Therefore, recent studies have been conducted to evaluate the relationship between herbal medicines and NAFLD. Some alternative and complementary medicines, such as ginger (Rahimlou et al., 2016), cinnamon (Askari et al., 2014), camelina sativa (Musazadeh, Dehghan, & Khoshbaten, 2022), and curcumin (White & Lee, 2019), have been studied for their use in NAFLD.

Turmeric, a member of the Zingiberaceae family, is the powdered rhizome of *Curcuma longa* (Maheshwari et al., 2006). Curcuminoids, the main ingredients of turmeric extract, are considered safe and beneficial compounds for various diseases (Kalhori et al., 2022). For example, turmeric has positive effects on the management of metabolic syndrome, anxiety, hyperlipidemia, arthritis, and oxidative and inflammatory conditions (Hewlings & Kalman, 2017; Musazadeh, Golandam, et al., 2022; Musazadeh, Roshanravan, et al., 2022; Naghsh et al., 2023). Moreover, it has been shown that curcumin possesses therapeutic advantages when it comes to oral mucosal diseases, periodontal diseases, and mouth neoplasms (Tang et al., 2021). Furthermore, a lower dose of curcumin may provide effective benefits for healthy individuals (Hewlings & Kalman, 2017). Some studies have shown that curcumin supplementation induces a reduction in AST levels in patients with NAFLD (Chashmniam et al., 2019; Panahi et al., 2017; Saadati, Sadeghi, et al., 2019), while others have failed to find this relationship (Hariri et al., 2020; Saberi‐Karimian et al., 2020). Similarly, conflicting results regarding the effects of curcumin supplementation on ALT levels in NAFLD patients have also been reported (Saadati, Sadeghi, et al., 2019; Saberi‐Karimian et al., 2020). For instance, a randomized clinical trial (RCT) found that curcumin administration at doses of 1500 mg/day over 12 weeks led to a significant reduction in ALT compared to placebo administration (Saadati, Hatami, et al., 2019), while another RCT did not find a significant association between curcumin supplementation and ALT in these patients (Saberi‐Karimian et al., 2020). Pooled results from a systematic review and meta‐analysis of RCTs indicated that curcumin had a significant reducing effect on AST and ALT in patients with NAFLD (Jalali et al., 2020).

Despite the various beneficial effects of curcumin and its health‐promoting properties, there is limited systematic review and meta‐analysis considering the effects of curcumin on liver enzymes or ALP. Thus, the aim of the present systematic review and meta‐analysis was to evaluate the current available literature and RCTs regarding the effects of curcumin supplementation on liver enzymes and ALP among patients with NAFLD.

## 2 METHODS

### 2.1 Search strategy

The present systematic review and meta‐analysis of RCTs were conducted using 2020 Preferred Reporting Items for Systematic Reviews and Meta‐Analyses (PRISMA) guidelines to assess the association between curcumin supplementation and liver enzymes (AST and ALT) and ALP in adults with NAFLD. All studies published up to November 18, 2022, were searched through the PubMed, SCOPUS, and Web of Science databases. The used keywords in this study are shown in File S1. No language restriction was applied in this systematic review and meta‐analysis. We also checked the reference lists of previous systematic reviews to avoid missing any studies.

### 2.2 Inclusion criteria

The population, intervention, comparison, outcome, and study design (PICOS) criteria were used as the inclusion criteria in this systematic review (Table 1). Parallel RCTs investigating the effects of curcumin or its derivatives supplementation on liver enzymes (AST and ALT) and ALP in patients with NAFLD were included.

**TABLE 1:** The inclusion criteria of PICOS.

PICOS components	Detail
Participants	Adults average age > 18 years with NAFLD.
Interventions	Curcumin supplementation
Comparisons	Placebo
Outcomes	AST, ALT, and ALP
Study designs	RCTs

### 2.3 Exclusion criteria

Studies were excluded if they met the following criteria: (1) studies conducted on cells, (2) studies conducted on animals, (3) studies conducted on pregnant or lactating women, and (4) studies conducted on children. Additionally, studies were excluded if they had the following characteristics: (5) measurements of liver enzymes immediately after curcumin supplementation, (6) absence of a placebo group, and (7) dissertations and conference abstracts. Studies with insufficient information were also excluded from this systematic review and meta‐analysis.

### 2.4 Data extraction and quality assessment

Two authors (AM and SK) independently reviewed the studies for eligibility and extracted the following information: first author's name, publication year, country, participants' gender and age, study design, curcumin dosage, study duration, outcomes of interest, outcome assessment methods, mean and standard deviation (SD) for ALT and AST at baseline and end of treatment, or changes in these measurements throughout the study.

The methodological quality of the included studies was evaluated using the Cochrane criteria for systematic review of interventions. AE assessed the risk of bias, considering random sequence generation, allocation concealment, blinding of participants and personnel, blinding of outcome assessors, completeness of outcome data, selectivity of outcome reporting, and other biases. The risk of bias in each study was categorized as low, high, or unclear (Higgins & Green, 2011). Furthermore, the evidence was assessed and ranked based on the grading of recommendation assessment, development, and evaluation (GRADE) approach for high risk of bias, inconsistency, imprecision, indirectness, and publication bias (Andrews et al., 2013).

### 2.5 Data synthesis and statistical analysis

Changes in the mean and 95% confidence intervals (CIs) of liver enzymes, including ALT and AST, in the intervention and control groups, were used to calculate the overall weighted mean difference (WMD) and 95% CI using the random‐effects model. Cochran's Q test and I‐squared statistics were used to evaluate the between‐study heterogeneity (significant heterogeneity was considered when *I* ^2^ was greater than 50%). Furthermore, subgroup analysis was conducted to identify possible sources of heterogeneity. Fixed‐effect analysis was used for all subgroup analyses. A sensitivity analysis was performed to determine if any specific study had a significant effect on the final findings. Publication bias was assessed through visual inspection of funnel plots and confirmed using Egger's regression test. STATA version 11.0 was utilized for all statistical analyses.

## 3 RESULTS

### 3.1 Characteristics of the included studies

**FIGURE 1:**
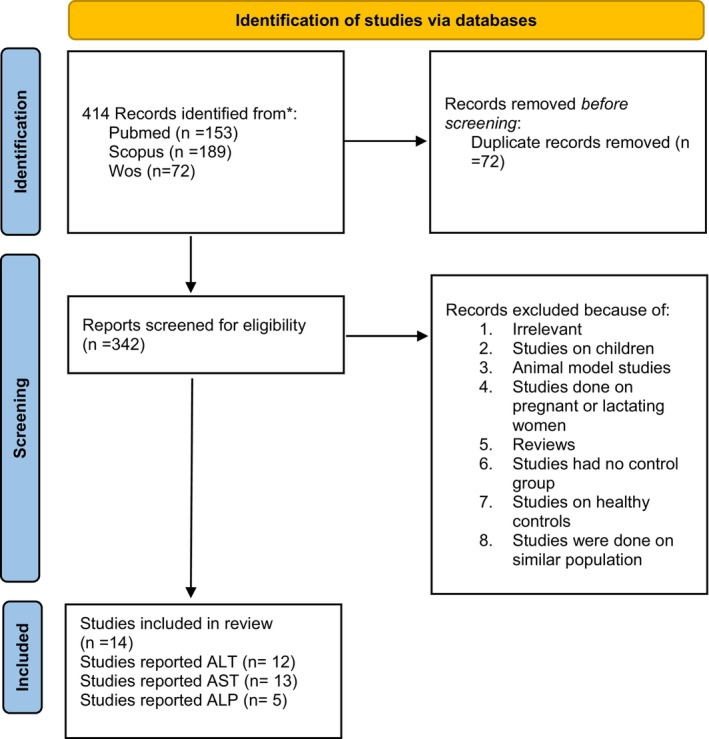
Literature search and review flowchart.

In total, 414 articles were retrieved by searching PubMed, Scopus, and Web of Science. After removing duplicates, 342 articles remained for screening based on the title/abstract and for detailed evaluation. Then, studies were excluded based on the following exclusion criteria: irrelevance, studies on children, animal model studies, studies conducted on pregnant or lactating women, reviews, studies without a control group, and studies on healthy controls (Figure 1). Finally, 14 articles were included in this systematic review (Table 2). These 14 articles were published between 2016 and 2021. A total of 778 patients with NAFLD of both sexes (394 in the intervention groups and 384 in the placebo groups), with a mean age ranging between 40.95 and 66.72 years, were included in this systematic review. Curcumin was administered at doses ranging from 50 to 3000 mg per day in the included studies for 8 to 12 weeks.

**TABLE 2:** General characteristics of included studies.

Id	Author/year	Country	Subject and gender	Age range and mean	Design	Curcumin dosage/day	Duration (week)	Outcomes	Outcome of assessment method	Outcome		Disease	References
										Placebo	Intervention		
1	Chashmniam et al. (2019)	Iran	I (n=25) gender (M/F): 13/12 P (n=20) gender (M/F): 14/6	I: 46.56 ± 2.25 P: 37.75 ± 3.22	Parallel	a capsule of phospholipid curcumin with a dose of 250 mg/day (Meriva®; each capsule composed of 250 mg/day, which was equivalent to 50 mg/day pure curcumin)	8 weeks	AST (U/L) ALP (IU/L)	Blood samples were centrifuged at 3000 rpm for 10 min and serum was separated and biochemical measurements were performed immediately after sampling using the BT‐2000 AutoAnalyzer machine (Biotechnica, Rome, Italy) through clinical routine methods. Residual serum was stored in aliquots at –80°C until metabolomics analyses were performed	AST B: 24.6 ± 11.14 A: 29.5 ± 8.54 ALP B: 184.95 ± 52.59 A: 176.8 ± 46.82	AST B: 35.16 ± 19.5 A: 31.85 ± 17.05 ALP B: 206 ± 72.25 A: 197.6 ± 71.3	NAFLD	Chashmniam et al. (2019)
2	Hariri et al. (2020)	Iran	I (*n* = 23) M (*n*) (%): 14 (53) F (*n*) (%): 9 (47) P (*n* = 22) M (*n*) (%): 12 (47) F (*n*) (%): 10 (53)	I: 40.95 (12.24) P: 40.06 (13.69)	Parallel	Phytosomal curcumin group (Meriva, 250 mg phospholipidated curcumin equivalent to 50 mg curcumin)	8 weeks	ALT (IU/L) AST (IU/L)	All biochemical variables were measured in the same laboratory, and standard laboratory methods were used for all samples. Liver enzymes (alanine transaminase (ALT) and aspartate aminotransferase (AST)) were measured using auto analyzer (BT‐2000) (Pars Azmoon, Tehran, Iran) once at baseline and once after eight weeks of intervention	ALT (IU/l) B: 38.91 (20.86) A: 38.45 (17.73) AST (IU/l) B: 26.66 (10.65) A: 27.76 (11.76)	ALT (IU/l) B: 45.59 (28.79) A: 42.77 (22.63) AST (IU/l) B: 32.01 (16.43) A: 29.97 (16.05)	NAFLD	Hariri et al. (2020)
3	Mirhafez et al. (2019)	Iran	I: *N* = 32 M 18 (48.6) F 14 (63.6) P: *N* = 27 M 19 (51.4) F 8 (36.4)	I: 44.8 (11.14) P: 40.7 (11.83)	Parallel	Phospholipidated curcumin (Meriva; each capsule composed of 250 mg/day equivalent to 50 mg/day pure curcumin)	8 weeks	AST (mg/dL) ALT (mg/dL)	Laboratory tests for liver enzymes (ALT and AST) were measured with an autoanalyzer (BT‐2000) (Pars Azmoun; Iran)	AST B: 25.18 (11.11) A: 27.62 (9.89) ALT B: 40.07 (19.82) A: 41.29 (19.29)	AST B: 32.45 (18.60) A: 30.45 (15.61) ALT B: 47.66 (35.20) A: 41.54 (22.98)	NAFLD	Mirhafez et al. (2019)
4	Moradi Kelardeh et al. (2020)	Iran	*N* = 22 I: 11 P: 11	I: 66.72 ± 3.03 P: 64.36 ± 2.97	Parallel	80 mg per day (Curcumin 80 mg as Nanomicelle produced by Minoo Pharmaceutical Co.)	12 weeks	ALP (IU/L)	ALP levels was measured by using biochemical analysis kits (Pars Azmoon Chemical co., Iran)	ALP B: 387.36 ± 79.70 A: 387.81 ± 74.75	ALP B: 376.36 ± 88.45 A: 376.09 ± 86.70	NAFLD	Moradi Kelardeh et al. (2020)
5	Navekar et al. (2017)	Iran	I (*n* = 21) P (*n* = 21) M: I: (*n* = 10) 52.4% P: (*n* = 8) 38.1% F I: (*n* = 11) 47.6% P: (*n* = 13) 61.9%	I: 42.09 (7.23) P: 40.38 (9.26)	Parallel	3 g/day 6 turmeric capsules daily. Each capsule contained 500 mg turmeric powder (6 £ 500 mg)	12 weeks	AST (IU/L) ALT (IU/L)	ALT and AST were measured using the standard enzymatic methods with a commercially available Pars Azmoon kit, Karaj, Iran)	AST B: 24.33 (13.69) A: 24.04 (5.4) ALT B: 45.585 (28.75) A: 28.925 (12.5)	AST B: 24.00 (11.59) A: 24.14 (8.9) ALT B: 41.785 (25.75) A: 25.185 (10.25)	NAFLD	Navekar et al. (2017)
6	Panahi et al. (2019)	Iran	I (*N* = 35) F: 15 (42.9%) M: 20 (57.1%) P (*N* = 35) F: 16 (45.7%) M: 19 (55.7%)	I: 46.63 ± 2.21 P: 47.51 ± 2.45	Parallel	500 mg/day Curcumin capsules (C3 Complex, Sami Labs Ltd, Bangalore, India) C3 Complex preparation that was used in the present study contains the three major curcuminoids including curcumin, demethoxycurcumin and bisdemethoxycurcumin in patented ratio	12 weeks	ALT, U/L AST, U/L ALP, U/L	Serum levels of alanine aminotransferase (ALT), aspartate aminotransferase (AST), alkaline phosphatase (ALP), were measured at baseline and at the end of study using routine enzymatic assays with commercial kits (Pars Azmoon, Tehran, Iran)	ALT B: 34.33 ± 27.44 A: 31.33 ± 15.45 AST B: 31.82 ± 13.94 A: 26.14 ± 11.99 ALP B: 194.39 ± 101.17 A: 188.91 ± 72.75	ALT B: 46.33 ± 32.46 A: 29.66 ± 9.27 AST B: 34.26 ± 21.45 A: 24.78 ± 12.41 ALP B: 196.16 ± 40.19 A: 160.75 ± 47.73	NAFLD	Panahi et al. (2019)
7	Rahmani et al. (2016)	Iran	I: *N* = 40 (M/F) 19/21 P: *N* = 40 (M/F) 19/21	I: 46.37 ± 11.57 P: 48.95 ± 9.78	Parallel	500 mg/day (500 mg/day of an amorphous dispersion preparation comprising 70‐mg curcuminoids)	8 weeks	ALT mg/dL AST mg/dL	ALT and AST were measured at baseline and at the end of study using routine enzymatic assays with commercial kits (Pars Azmoon, Tehran, Iran)	ALT B: 30.35 ± 13.97 A: 28.72 ± 10.93 AST B: 32.05 ± 17.64 A: 34.07 ± 18.73	ALT B: 39.07 ± 19.79 A: 36.08 ± 46.58 AST B: 28.88 ± 10.60 A: 23.84 ± 7.83	NAFLD	Rahmani et al. (2016)
8	Saberi‐Karimiana et al. (2020)	Iran	I: *N* =27 P: *N* = 28	18–70 years	Parallel	500 mg/day 500 mg curcuminoids (plus 5 mg piperine to increase intestinal absorption)	8 weeks	ALT (U/L) AST (U/L)	alanine aminotransferase (ALT) and aspartate aminotransferase (AST) activities were assessed using Pars Azmoon kits (Tehran, Iran)	ALT 30.40 ± 16.14 Changes −4.72 ± 10.74 AST 24.55 ± 8.41 Changes −1.08 ± 5.19	ALT 30.00 ± 15.12 Changes −0.18 ± 12.75 AST 24.70 ± 6.90 Changes 0.13 ± 7.09	NAFLD	Saberi‐Karimian et al. (2020)
9	Saadati, Hatami, et al. (2019)	Iran	I: *N* = 27 M: 13 (48.1%) F: 14 (60.9%) P: *N* = 23 M: 14 (51.9%) F: 9 (%39.1)	I: 46.19 ± 11.5 P: 45.13 ± 10.9	Parallel	1500 mg/day (Curcumin capsules were filled withBCM‐95 (BIO‐CURCUMIN®), a proprietary combination of 95% curcuminoids and essential oil of turmeric‐ar‐turmerone)	12 weeks	ALT (IU/L) AST (IU/L)	AST and ALT were assessed by enzymatic methods using Pars azmun kits (Parsazmun Co., Tehran, Iran)	ALT (IU/L) B: 28.02 ± 13.06 A: 21.2 ± 7.72 AST (IU/L) B: 16.23 ± 5.66 A: 12.77 ± 4.05	ALT (IU/L) B: 26.05 ± 15.09 A: 20.42 ± 10.46 AST (IU/L) B: 17.65 ± 9.95 A: 14.93 ± 7.6	NAFLD	(Saadati et al. 2019)
10	Jazayeri‐Tehrani et al. (2019)	Iran	I (*n* = 42) Male: 23 (54.8) Female: 19 (45.2) Placebo (*n* = 42) Male: 23 (54.8) Female: 19 (45.2)	I: 41.8 (5.6) P: 42.5 (6.2)	Parallel	80 mg/day (two 40‐mg capsules per day	12 weeks	ALT (u/L) AST (u/L)	Blood sampling, storage, and laboratory tests were carried out at the NIOC Central Hospital, Tehran. AST: Bionik®, Liquid Stable, NADH. Kinetic UV.Liquid, Intra‐assay CV ≤ 3.02%, Inter‐assay CV ≤ 3.00% ALT: Bionik®, Liquid Stable, NADH. Kinetic UV.IFCC, Intra‐assay CV ≤ 4.27%, Inter‐assay CV ≤ 4.68%	ALT (u/L) B: 42.1 (8.2) A: 39.6 (7.5) AST (u/L) B: 27.60 (7.8) A: 25.63 (7.2)	ALT (u/L) B: 42.8 (11.6) A: 32.6 (9.9) AST (u/L) B: 28.43 (6.7) A: 22.03 (5.9)	Overweight/obese patients with NAFLD	Jazayeri‐Tehrani et al. (2019)
11	Jarhahzadeh et al. (2021)	Iran	I (*n* = 32) (M/F): 19/13 P (*n* = 32) (M/F): 19/13	I: 44.12 ± 8.35 P: 38.56 ± 10.43	Parallel	Turmeric (2 g/daily)	8 weeks	ALT (U/L) AST (U/L)	Hematological factors including ALT and AST were determined by an automated biochemical analyzer (Hitachi‐7180E, Tokyo, Japan) with a Pars Azmoon reagent kit (Tehran, Iran)	ALT (U/L) B: 35.42 ± 18.51 A: 39.50 ± 21.15 AST (U/L) B: 27.29 ± 11.53 A: 25.26 ± 9.66	ALT (U/L) B: 39.56 ± 22.41 A: 30.51 ± 12.61 AST (U/L) B: 26.81 ± 10.54 A: 21.19 ± 5.67	NAFLD	Jarhahzadeh et al. (2021)
12	Panahi et al. (2017)	Iran	I: 44 F (%): 20 (45.5) P: 43 F (%): 16 (37.2)	I: 44.98 ± 12.59 P: 47.21 ± 10.29	Parallel	1000 mg/day Curcumin (1000 mg/day in 2 divided doses) (*n* = 50) or control (*n* = 52) group. Curcumin was administered in the form of 500 mg capsules, and patients were advised to take the capsules after meal. Administered curcumin had a phytosomal formulation (Meriva®; Indena S.p.A, Milan, Italy) that contained a complex of curcumin and soy phosphatidylcholine in a 1:2 weight ratio, and 2 parts of microcrystalline to improve flowability, with an overall content of curcumin in the final product of around 20%	8 weeks	ALT (U/L) AST (U/L) ALP (U/L)	Serum levels of ALT, AST and ALP were measured at baseline and at the end of study using routine enzymatic assays with commercial kits (Pars Azmoon, Tehran, Iran)	ALT (U/L) B: 36.81 ± 24.32 A: 41.33 ± 23.97 AST (U/L) B: 27.44 ± 10.01 A: 31.23 ± 12.80 ALP (U/L) B: 170.77 ± 40.80 A: 172.59 ± 43.37	ALT (U/L) B: 35.46 ± 22.97 A: 24.85 ± 12.84 AST (U/L) B: 27.63 ± 11.35 A: 20.68 ± 6.65 ALP (U/L) B: 172.52 ± 40.65 A: 172.91 ± 39.85	NAFLD	Panahi et al. (2017)
13	Pakzad et al. (2019)	Iran	I: 50 F: 21 M: 29 P: 50 F: 20 M: 30	I: 44.26 ± 8.61 P: 46.28 ± 10.13	Parallel	50 mg	8 weeks	ALP ALT AST	NR	ALP B: 282.44 ± 21.34 A: 282.48 ± 21.62 ALT B: 83.74 ± 23.13 A: 81.80 ± 11.86 AST B: 86.12 ± 22.45 A: 79.78 ± 5.50	ALP B: 285.52 ± 22.05 A: 285.78 ± 21.35 ALT B: 86.80 ± 26.20 A: 36.68 ± 5.56 AST B: 92.96 ± 26.02 A: 37.38 ± 6.18	NAFLD	Pakzad et al. (2019)
14	Ghaffari et al. (2018)	Iran	I (*n* = 21) M: 11 (52.4%) F: 10 (47.6%) P (*n* = 21) M: 8 (38.1%) F: 13 (61.9%)	I: 42.5 ± 6.93 P: 40.3 ± 9.26	Parallel	3 g/day turmeric (1000 mg three times a day with meals)	12 weeks	AST (U/L) ALT (U/L)	Serum ALT and AST were measured by enzymatic method using a commercial kit (Pars Azmoon, Tehran, Iran)	AST (U/L): B: 24.3 ± 13.6 A: 24.0 ± 5.40 ALT (U/L): B: 45.55 (28.75) A: 28.9 (12.5)	AST (U/L): B: 24 ± 11.5 A: 24.1 ± 8.90 ALT (U/L): B: 41.75 (25.75) A: 25.15 (10.25)	Patients with Body Mass Index (BMI) ranged from 24.9 to 40 kg/m2 were included‐ NAFLD	Ghaffari et al. (2018)

### 3.2 Risk of bias assessment

The assessment of study quality is shown in Table 2. The risk of bias in the included studies was assessed using the Cochrane criteria, as shown in Table 3. Additionally, the GRADE approach was used to grade the quality of evidence for outcomes in Table 4. The quality of evidence for ALT, AST, and ALP was graded as “moderate” after downgrading for inconsistency. The details of the included RCTs are indicated in Table 2.

**TABLE 3:** (A) Assessment of risk of bias in the included studies using. (B) Grade profile of curcumin supplementation on liver enzymes.

ID	Study	Sequence generation	Allocation concealment	Blinding of participants and personnel	Blinding of outcome assessment	Incomplete outcome data	Selective outcome reporting	Other potential threats to validity	General risk of bias
1	Chashmniam et al. (2019)	L	L	L	L	L	L	L	L
2	Hariri et al. (2020)	L	L	L	L	L	L	L	L
3	Mirhafez et al. (2021) (a)	L	L	H	L	L	L	L	L
4	Moradi Kelardeh et al. (2020)	L	U	L	L	L	L	U	L
5	Navekar et al. (2017)	L	L	L	U	L	L	L	L
6	Panahi et al. (2019)	U	L	L	U	L	L	L	L
7	Rahmani et al. (2016)	L	L	L	L	L	L	L	L
8	Saberi‐Karimiana et al. (2020)	L	L	L	L	L	L	L	L
9	Saadati, Hatami, et al. (2019)	L	L	L	L	L	L	L	L
10	Jazayeri‐Tehrani et al. (2019)	L	L	L	L	L	L	L	L
11	Jarhahzadeh et al. (2021)	U	U	U	L	U	L	L	L
12	Panahi et al. (2017)	U	L	L	L	L	L	L	L
13	Pakzad et al. (2019)	L	L	L	L	L	L	U	L
14	Ghaffari et al. (2018)	L	L	L	L	L	L	U	L

Quality assessment						Summary of findings	Quality of evidence
Outcomes	Risk of bias	Inconsistency	Indirectness	Imprecision	Publication bias	WMD, (95% CI)	
ALT	No series limitation	Very series limitation	No series limitation	No series limitation	No series limitation	−8.72 (−15.16, −2.27)	⊕⊕⊕◯ Moderate
AST	No series limitation	Very series limitation	No series limitation	No series limitation	No series limitation	−6.35 (−9.81, −2.88)	⊕⊕⊕◯ Moderate
ALP	No series limitation	Series limitation	No series limitation	No series limitation	No series limitation	−4.71 (−13.01, 3.58)	⊕⊕⊕◯ Moderate

**TABLE 4:** Grade profile of curcumin supplementation on liver enzymes.

Variables	Subgroups		Number of effect sizes	Pooled WMD	95% CI	*I* ^2^ (%)	Between‐ study *I* ^2^ (%)
ALT	Age of participants	More than 45 years	3	−1.05	−4.46, 2.37	74.9	94.1
		Less than 45 years	8	−10.09	−11.60, −8.58	94.9	
	Dosage of curcumin therapy	Less than 1000 mg/day	8	−9.26	−10.77, −7.74	95.4	94.1
		More than 1 00 mg/day	4	−3.30	−6.23, 0.36	84	
	Duration of curcumin therapy	8 weeks	7	−12.24	−14.61, −9.86	95.9	94.1
		More than 8 weeks	5	−6	−7.64, −4.37	80.7	
	Sample size of studies	Less than 55 peoples	5	1.06	−1.71, 3.83	0	94.1
		More than 55 peoples	7	−10.8	−12.34, −9.26	95.3	
AST	Age of participants	More than 45 years	4	−2.84	−4.3, −1.38	88.8	94.5
		Less than 45 years	8	−5.43	−6.37, −4.48	95.8	
	Dosage of curcumin therapy	Less than 1000 mg/day	9	−5.83	−6.74, −4.92	95.3	94.4
		More than 1000 mg/day	4	−0.56	−2.01, 0.89	55.3	
	Duration of curcumin therapy	8 weeks	8	−6.59	−7.75, −5.43	95.8	94.4
		More than 8 weeks	5	−2.55	−3.58, −1.51	81	
	Sample size of studies	Less than 55 peoples	6	−0.72	−2.05, 0.60	73.5	94.4
		More than 55 peoples	7	−6.19	−7.14, −5.24	96	

ALP	Dosage of curcumin therapy	Less than 500 mg/day	3	0.18	−3.48, 3.85	0	64.2
		More than 500 mg/day	2	−6.24	−13.33, 0.86	88.5	
	Duration of curcumin therapy	8 weeks	3	−0.1	−3.44, 3.23	0	64.2
		12 weeks	2	−23.01	−38.1, −7.91	61.6	
	Sample size of studies	Less than 55 peoples	2	−0.35	−14.64, 13.94	0	64.2
		More than 55 peoples	3	−1.21	−4.56, 2.13	82.1	

### 3.3 Findings for the effects of curcumin administration on ALT

**FIGURE 2:**
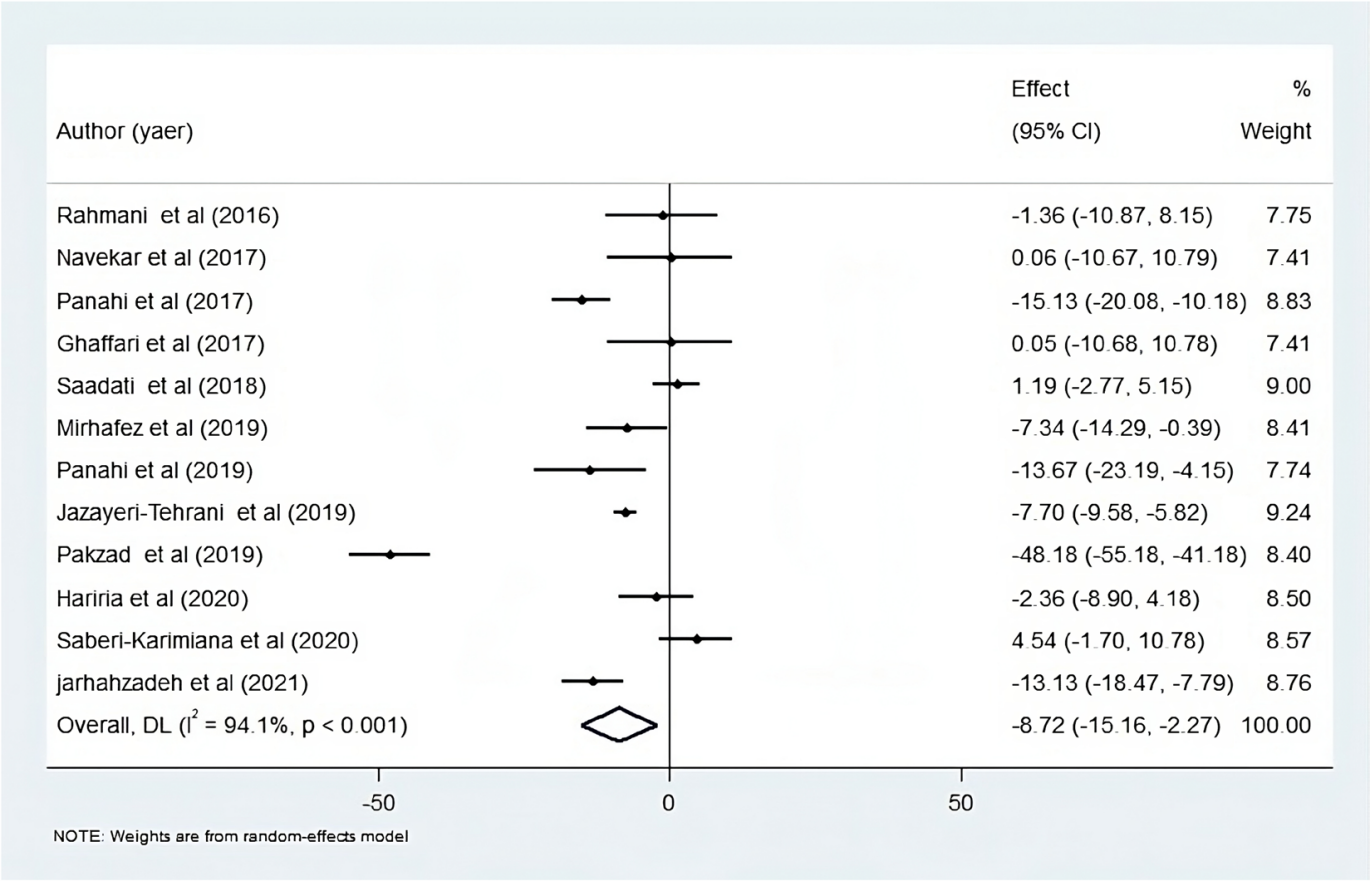
Forest plot for the effect of curcumin therapy on ALT in NAFLD patients; expressed as the mean differences between the intervention and the control. The area of each square is proportional to the inverse of the variance of the WMD. Horizontal lines represent 95% of CIs. Diamonds represent pooled estimates from random‐effects analysis.

In this study, the pooled meta‐analysis indicated that curcumin supplementation at doses of 50–3000 mg/day over 8–12 weeks could significantly reduce ALT in the intervention group compared to the placebo group. This analysis included 12 articles with 12 effect sizes (Figure 2) (Ghaffari et al., 2017; Hariri et al., 2020; Jarhahzadeh et al., 2021; Jazayeri‐Tehrani et al., 2019; Mirhafez et al., 2019; Mirhafez et al., 2021; Navekar et al., 2017; Pakzad et al., 2019; Panahi et al., 2017; Panahi et al., 2019; Rahmani et al., 2016; Saadati, Hatami, et al., 2019; Saadati, Sadeghi, et al., 2019; Saberi‐Karimian et al., 2020). Due to high heterogeneity among the studies, subgroup analyses were conducted based on participants' age (age ≤45 years and >45 years), curcumin dosage (≤1000 mg/day and >1000 mg/day), study duration (8 and 12 weeks), and study sample size (*n* ≤ 55 and *n* > 55). The results of the subgroup analyses indicated that curcumin supplementation could significantly decrease ALT in patients younger than 45 years, while this association was not observed in patients older than 45 years. There was no difference in the effects of curcumin supplementation between studies of different durations and doses. Considering the duration of studies, Curcumin supplementation could decrease ALT levels in studies with both 8‐ and 12‐week durations. Furthermore, a significant reduction in ALT was observed in studies with larger sample sizes (more than 55 patients) following curcumin consumption (Table 5). Sensitivity analysis showed that no individual study had a major influence on the overall results. Additionally, no publication bias was found in the included studies (P‐Egger test = 0.31 and P‐Begg's test = 0.38).

**TABLE 5:** Subgroup analyses for the effects of curcumin therapy on ALT, AST, and ALP.

Variables	Subgroups		Number of effect sizes	Pooled WMD	95% CI	*I* ^2^ (%)	Between‐study *I* ^2^ (%)
ALT	Age of participants	More than 45 years	4	−0.18	−2.82, 2.46	65.1	93.6
		Less than 45 years	9	−10.01	−11.50, −8.52	94.2	
	Dosage of curcumin therapy	Less than 1000 mg/day	9	−9.19	−10.69, −7.69	94.8	93.6
		More than 1000 mg/day	5	−1.84	−4.24, 0.56	81.4	
	Duration of curcumin therapy	8 weeks	8	−11.95	−14.27, −9.63	95.3	93.6
		12 weeks	6	−5.06	−6.58, −3.54	83.6	
	Sample size of studies	Less than 55 peoples	7	0.78	−1.47, 3.04	0	93.6
		More than 55 peoples	7	−10.8	−12.34, −9.26	95.3	
AST	Age of participants	More than 45 years	5	−1.72	−2.9, −0.54	88	94.2
		Less than 45 years	9	−5.38	−6.31, −4.46	95.2	
	Dosage of curcumin therapy	Less than 1000 mg/day	10	−5.78	−6.67, −4.88	94.7	94
		More than 1000 mg/day	5	−0.22	−1.4, 0.95	45.4	
	Duration of curcumin therapy	8 weeks	9	−6.45	−7.58, −5.33	95.2	94
		12 weeks	6	−1.93	−2.85, −1.01	82	
	Sample size of studies	Less than 55 peoples	8	−0.61	−1.68, 0.47	69.1	94
		More than 55 peoples	7	−6.19	−7.14, −5.24	96	
ALP	Dosage of curcumin therapy	Less than 500 mg/day	3	0.18	−3.48, 3.85	0	64.2
		More than 500 mg/day	2	−6.24	−13.33, 0.86	88.5	
	Duration of curcumin therapy	8 weeks	3	−0.1	−3.44, 3.23	0	64.2
		12 weeks	2	−23.01	−38.1, −7.91	61.6	
	Sample size of studies	Less than 55 peoples	2	−0.35	−14.64, 13.94	0	64.2
		More than 55 peoples	3	−1.21	−4.56, 2.13	82.1	

### 3.4 Findings for the effects of curcumin administration on AST

**FIGURE 3:**
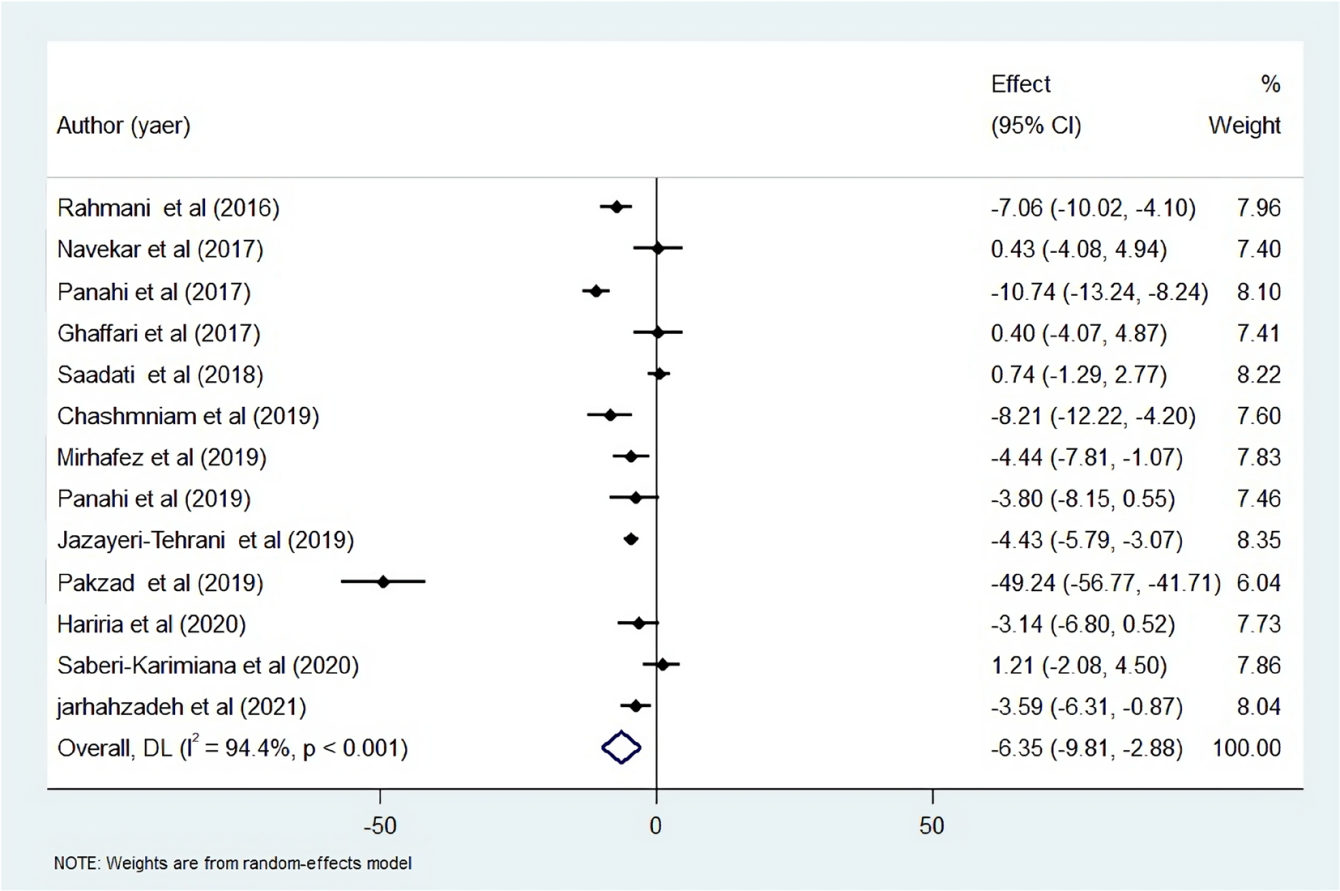
Forest plot for the effect of curcumin therapy on AST in NAFLD patients; expressed as the mean differences between the intervention and the control. The area of each square is proportional to the inverse of the variance of the WMD. Horizontal lines represent 95% of CIs. Diamonds represent pooled estimates from random‐effects analysis.

A pooled meta‐analysis of the included 13 studies showed that curcumin administration at doses of 50–3000 mg/day over 8–12 weeks could significantly decrease AST in the intervention group compared to the placebo group [weighted mean difference (WMD): −6.35; 95% CI: −9.81, −2.88, *I* ^2^ = 94.4%] (Figure 3) (Chashmniam et al., 2019; Ghaffari et al., 2017; Hariri et al., 2020; Jarhahzadeh et al., 2021; Jazayeri‐Tehrani et al., 2019; Mirhafez et al., 2019, 2021; Navekar et al., 2017; Pakzad et al., 2019; Panahi et al., 2017, 2019; Rahmani et al., 2016; Saadati, Hatami, et al., 2019; Saadati, Sadeghi, et al., 2019; Saberi‐Karimian et al., 2020). The same subgroup analyses conducted for ALT were also performed for AST to identify the source of high heterogeneity among the studies. No differences were observed between age groups. Subgroup analysis indicated that curcumin supplementation could significantly reduce AST in both age groups of patients. Although curcumin administration at low doses (less than 1000 mg/day) was significantly associated with a reduction in AST, higher doses of curcumin (more than 1000 mg/day) were not. Furthermore, no differences were seen between studies with varying durations in terms of the effects of curcumin therapy. Curcumin administration could decrease AST in studies with both 8 and 12 weeks of duration. Subgroup analysis based on the sample size of the studies indicated that a significant reduction in AST was seen in the studies with sample sizes larger than 55 participants (Table 5). No evidence was found for a significant influence of any individual study on the overall findings. We found publication bias in the included studies according to Egger's test (*p* < .001), but not according to Begg's test (*p* = .62). Thus, the trim‐and‐fill test was conducted to correct the bias; however, no additional studies were suggested by the test.

### 3.5 Findings for the effects of curcumin administration on ALP

**FIGURE 4:**
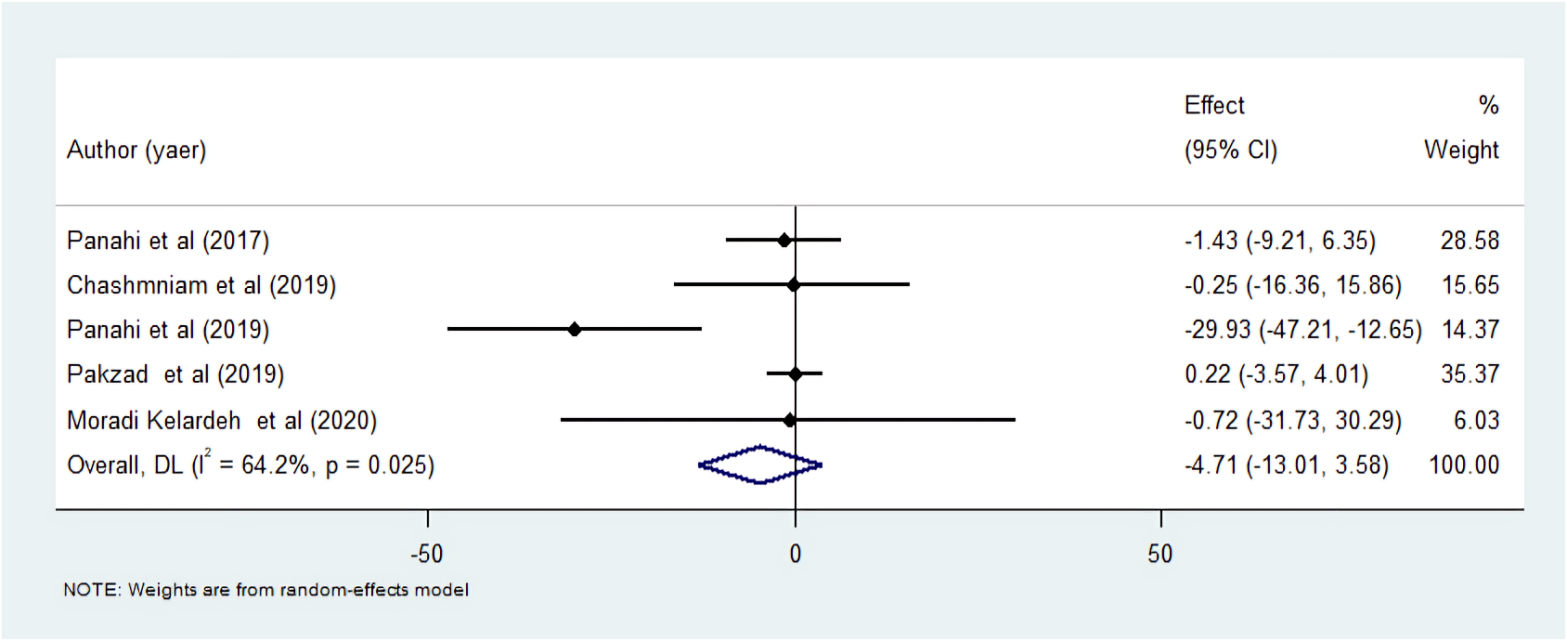
Forest plot for the effect of curcumin therapy on ALP in NAFLD patients; expressed as the mean differences between the intervention and the control. The area of each square is proportional to the inverse of the variance of the WMD. Horizontal lines represent 95% of CIs. Diamonds represent pooled estimates from random‐effects analysis.

A pooled analysis of five trials indicated that there was no significant association between curcumin therapy and ALP in NAFLD patients (Figure 4) (Chashmniam et al., 2019; Ghaffari et al., 2018; Moradi Kelardeh et al., 2020; Pakzad et al., 2019; Panahi et al., 2017, 2019). Subgroup analysis was conducted considering the dosage (less than and greater than 500 mg/day) and duration (8 and 12 weeks) of curcumin therapy, as well as the sample sizes (less than and greater than 55 people) of the included studies. Subgroup analysis considering the dose of supplementation showed that there was no difference between studies with various doses about the effects of curcumin on ALP. Additionally, curcumin therapy for 12 weeks could significantly affect ALP, while 8 weeks of supplementation could not affect it significantly. However, subgroup analysis regarding the sample size of the included studies did not show any difference in the effects of various studies with different sample sizes (Table 5). We found no evidence for publication bias (P‐Egger test = 0.97 and P‐Begg's test = 0.25) and no significant influence of any individual study on the overall results.

## 4 DISCUSSION

This systematic review and meta‐analysis aimed to review the available literature and randomized controlled trials (RCTs) considering the effects of curcumin supplementation on liver enzymes, including ALT, AST, and ALP. The present meta‐analysis indicated a significant association between curcumin supplementation and a decrease in ALT and AST levels in patients with nonalcoholic fatty liver disease (NAFLD). However, no significant changes were observed in ALP levels following curcumin supplementation.

A significant decrease was observed in ALT after curcumin therapy in patients with nonalcoholic fatty liver disease (NAFLD). This finding was in line with a recent trial in patients with NAFLD, which showed that 250 mg/day of curcumin therapy for 8 weeks could cause a significant reduction in ALT (Mirhafez et al., 2021). Results of other randomized controlled trials (RCTs) also indicated that curcumin therapy could decrease ALT in these patients (Panahi et al., 2019; Rahmani et al., 2016). However, Hariri et al. conducted a trial in 2020, and their study did not show a significant association between curcumin therapy and ALT in these patients (Hariri et al., 2020). Furthermore, a similar RCT indicated that curcumin therapy did not have an effective result on ALT in these patients (Navekar et al., 2017). One possible reason for this discrepancy in results could be related to the fact that Hariri et al.'s study did not consider high liver enzyme concentration as inclusion criterion. Another limitation was that the serum level of curcumin was not measured to evaluate the patients' adherence. Additionally, the sample size was relatively small in their study. Subgroup analysis by sample size in the present meta‐analysis showed that RCTs with a higher sample size were associated with a reduction in ALT. Furthermore, another RCT conducted on patients older than 45 years indicated that curcumin therapy could not significantly affect ALT (Saadati, Sadeghi, et al., 2019). Subgroup analysis in this meta‐analysis indicated that curcumin therapy had a significant effect on ALT in younger patients, while it was not effective in those older than 45 years. In line with this finding, a systematic review and meta‐analysis published in 2022 indicated that curcumin therapy had a reducing effect on ALT in patients with NAFLD (Ngu et al., 2022).

The current meta‐analysis indicates that curcumin therapy could significantly decrease AST in patients with nonalcoholic fatty liver disease (NAFLD). Several randomized controlled trials (RCTs) have reported similar results to the present study (Mirhafez et al., 2021; Panahi et al., 2019; Rahmani et al., 2016). For example, Panahi et al. conducted a study in 2019 and concluded that curcumin therapy has a reductive effect on AST in these patients (Panahi et al., 2019). However, other trials have reported contradictory results to this systematic review and meta‐analysis. A pilot study conducted in 2020 showed that curcumin supplementation could not change AST levels in these patients (Hariri et al., 2020). Additionally, another RCT in 2017 indicated that curcumin has no significant effect on AST (Navekar et al., 2017). The limited sample size in some of these studies could be one of the reasons for the discrepancy in results. However, the subgroup analysis conducted in the present meta‐analysis indicates that studies with a larger sample size are associated with a decrease in AST. Another systematic review and meta‐analysis conducted by Ngu, Norhayati et al. in 2022 also demonstrated that curcumin therapy can significantly decrease AST in patients with NAFLD (Ngu et al., 2022). However, it is important to note that the current systematic review and meta‐analysis included subgroup analysis considering various variables, while the previous meta‐analysis only conducted subgroup analysis based on the dosage of curcumin therapy.

The current meta‐analysis indicates that there is no relationship between curcumin therapy and ALP levels in patients with nonalcoholic fatty liver disease (NAFLD). This finding is consistent with a randomized controlled trial (RCT) conducted by Panahi, Kianpour et al. (Panahi et al., 2017), which showed that curcumin therapy did not have a significant effect on ALP in NAFLD patients. Similarly, another RCT did not demonstrate a significant association between curcumin therapy and ALP (Chashmniam et al., 2019). However, Panahi et al. indicated that curcumin administration significantly reduced ALP levels in NAFLD patients (Panahi et al., 2019). Possible reasons for the discrepancy among these studies could be attributed to the longer duration and higher dosage of curcumin therapy in Panahi et al.'s study compared to the other RCTs. Additionally, subgroup analysis in this meta‐analysis indicated that a longer duration of curcumin therapy was associated with significant improvements in ALP, while no changes were observed with shorter duration of curcumin therapy. One possible reason for the nonsignificant effects of curcumin therapy with a shorter duration on ALP could be the very poor bioavailability of curcumin (Lopresti, 2018). Curcumin concentrations in the blood and extraintestinal tissue are often very low or even undetectable due to its low absorption, poor bioavailability, chemical instability, rapid metabolism, and systemic elimination (Anand et al., 2007; Lopresti, 2018).

Considering the results of the current study, curcumin is a promising but not conclusive treatment for nonalcoholic fatty liver disease (NAFLD), and the exact mechanism of curcumin on liver enzymes is not clear. In a previous study, it was asserted that alanine aminotransferase (ALT) is considered a standard indicator of liver function that is related to hepatic inflammation and liver injury in patients with various chronic liver diseases (Ma et al., 2020). Elevated liver enzymes have also been shown to be markers of inflammation and oxidative stress (Yamada et al., 2006). The key role of tumor necrosis factor alpha (TNF‐α) in the pathogenesis of NAFLD has been observed before (Kakino et al., 2018). Curcumin, as a complementary and alternative therapy, has demonstrated antioxidant and anti‐inflammatory properties in many chronic diseases (He et al., 2015; Naghsh et al., 2023). Curcumin has the ability to decrease proinflammatory markers and suppress the production of TNF and its cell signaling in different types of cells (He et al., 2015). The reductive effect of curcumin on ALT and AST in NAFLD patients could possibly be explained by the antioxidant properties of curcumin.

To the best of our knowledge, this study is among the few meta‐analyses evaluating the results of curcumin therapy on ALT, AST, and ALP in NAFLD patients. The current meta‐analysis had some limitations. First, only randomized controlled trials (RCTs) were included. On the other hand, studies used various doses, age ranges, durations, and sample sizes, which caused heterogeneity among studies. Moreover, all studies were done in Iran, hence the results could not be possibly generalized to other populations and the results should be interpreted with caution. However, the present study had some strengths as well. One of the strengths of this study is the subgroup analysis based on participants' age, curcumin dosage, study duration, and sample size in order to reduce the effects of heterogeneity. On the other hand, all liver function tests were completely assessed following curcumin supplementation which could give us a good insight into the effects of curcumin on liver function, especially in NAFLD patients. Sensitivity analysis suggested that no individual study had an effect on the overall findings. Moreover, there was no evidence of publication bias among the included studies in this meta‐analysis.

In conclusion, the current systematic review and meta‐analysis indicate that curcumin therapy could potentially decrease ALT and AST in NAFLD patients, with no significant changes in ALP. However, further studies with larger sample sizes or different durations of curcumin therapy conducted in different regions are needed to draw a more definitive conclusion regarding the effects of curcumin supplementation on ALT, AST, and ALP in NAFLD patients. More trials investigating curcumin therapy and liver enzymes are also warranted to better elucidate the exact mechanism of action.

## AUTHOR CONTRIBUTIONS

**Armin Ebrahimzadeh:** Conceptualization (equal); data curation (equal); formal analysis (equal); software (equal); writing – review and editing (equal). **anahita ebrahimzadeh:** Writing – original draft (equal); writing – review and editing (equal). **sara fooladshekan:** Writing – original draft (equal); writing – review and editing (equal). **shokouh mohseni:** Data curation (equal); writing – original draft (equal). **Abbas Mohtashamian:** Data curation (equal); writing – original draft (equal). **Siavash Babajafari:** Conceptualization (equal); supervision (equal). **Zahra Sohrabi:** Conceptualization (equal); supervision (equal); writing – review and editing (equal).

## CONFLICT OF INTEREST STATEMENT

All authors report no conflicts of interest.

## Supporting information


File S1.

## Data Availability

Research data are not shared.
