# Supplementary material for: Therapeutic effects of curcumin supplementation on liver enzymes of nonalcoholic fatty liver disease patients: A systematic review and meta‐analysis of randomized clinical trials
Source: Food Sci Nutr. 2024 Dec 1;13(1):e4144. doi: 10.1002/fsn3.4144 (PMC11716989; doi:10.1002/fsn3.4144)
Supplement: Supplementary file 1 — File S1. [file FSN3-13-e4144-s001.docx]

Date: 2022/November/18

| Pubmed | | |
| --- | --- | --- |
| intervention | Population | number |
| “curcumin”[tiab] OR “curcumin”[MESH] OR “turmeric”[tiab] OR “Curcuma domestica”[tiab] OR “Curcuma Longa”[tiab] | "Non-alcoholic Fatty Liver Disease”[tiab] OR "Non-alcoholic Fatty Liver Disease”[MESH] OR "NAFLD”[tiab] OR "non-alcoholic steatohepatitis"[tiab] OR "non-alcoholic steatosis"[tiab] OR "non-alcoholic liver steatosis"[tiab] OR "non-alcoholic steatohepatitis"[tiab] OR "non-alcoholic steatosis"[tiab] OR "non-alcoholic hepatic steatosis"[tiab] OR "non-alcoholic liver steatosis"[tiab] OR "non-alcoholic hepatic steatosis"[tiab] | 153 |

| Scopus | | | |
| --- | --- | --- | --- |
| intervention | Population | RCT | number |
| TITLE-ABS-KEY ( “curcumin” OR “turmeric” OR “Curcuma domestica” OR “Curcuma Longa” ) | TITLE-ABS-KEY ( “Non-alcoholic Fatty Liver Disease” OR “Nonalcoholic Fatty Liver” OR “NAFLD” OR “nonalcoholic steatohepatitis” OR “nonalcoholic steatosis” OR “nonalcoholic steatohepatitis” ) | TITLE-ABS-KEY ( "randomized controlled trial" OR "clinical trial" OR "randomized" OR "clinical" OR "placebo" OR "trial" OR "randomly" OR "intervention" ) | 189 |

| Web of science | | |
| --- | --- | --- |
| intervention | Population | number |
| “curcumin” OR “turmeric” OR “Curcuma domestica” OR “Curcuma Longa” (Title) | “Non-alcoholic Fatty Liver Disease” OR “Nonalcoholic Fatty Liver” OR “NAFLD” OR “nonalcoholic steatohepatitis” OR “nonalcoholic steatosis” OR “nonalcoholic steatohepatitis” (Title) | 72 |
